# Quackery at Home and Abroad

**Published:** 1859-02

**Authors:** 


### EDITORIAL DEPARTMENT.

*Quackery at Home and Abroad.*—When our indulgent reader takes up this number of the Journal and sees the heading on this article, let him repress his inclination to turn to something more interesting than this well-worn theme. We would like to have a little familiar talk on medical topics, and would fain say something about quackery. Necessity compels us, however, to the course which some brilliant lights invariably adopt, that is to do all the talking ourselves. We will then begin by asking the question: Are we especially favored by Providence in the distribution of quacks; or are we greater sufferers than our brethren on the other side of the water? We opine that as a rule, the profession in this country suffers most; but occasionally we have accounts of such brilliant success of the gentry in Europe, that we feel almost inclined to yield the palm. An item has appeared in a late number of the Boston Journal which gives us some idea how the Parisians are sometimes humbugged, in the midst of their stringent regulations, in regard to the practice of Medicine. It has been said that the African race does not inspire the European with the same disgust which is felt for them by some of our philanthropic countrymen, and we therefore imagine that Paris would be an admirable field for the operations of a sable charlatan. We know that here, in the Southern States, it is not infrequent to find old negroes who have quite a celebrity in the treatment of various diseases; such a notoriety as is sometimes enjoyed by our old women in the North who have therapeutical tendencies and immense diagnostic intuition; but we have never before heard of a full-fledged black quack in successful practice. But for an example, see the following account of how Sambo is succeeding in the practice of Medicine in Paris.

*Quackery in Paris.**—There is now at Paris a negro who carries every-

---

**Appropos* of quackery in Paris, some of our readers may have heard of the libel suit against the *Union Médicale;* we clip the following, which gives the result of this remarkable suit, from a daily paper:

A curious libel suit has recently come off in Paris. Twelve homœopathic physithing before him as a quack Doctor. He is a fine man of his race, covered with trinkets and diamonds, displaying great wealth in house, carriages, &c., &c., and obtains the most fabulous fees from the easily-gulled Parisians. Amongst his various feats, *L'Union Médicale* relates the following. He was sent for the other day into a very rich family, where a lady had for years suffered from very obstinate recurrence of fibrous tumors about various parts of the body. The best surgeons of Paris had failed in arresting the disease, and recourse was naturally had to wild systems of medicine; all these, however, including homœopathy, were powerless. Magnetism, necromancy, &c., &c., had their turn, but nothing succeeded. At last, the negro's turn came. When he had cursorily examined the patient, he exclaimed, "this lady is curable, and I shall get her well in fourteen days." "Well, then," said the husband, "undertake the case at once." "My fee is £800; £240 is to be paid at once, and £40 on every other visit." Much demur was made to such a demand; but as the quack threatened to leave the lady to her fate, he was allowed to pocket the £240, and has begun the treatment. The result is not known, but may easily be guessed at.—*Druggists' Circular.*

We have never thought of this before, but it now strikes us what immense natural advantages the black race has over the white in the field of quackery. Once out of the reach of the prejudice which would prevent success among *intelligent people*, in this country, all his national characteristics operate immensely in his favor. First among the essential elements of his calling, is *unblushing* effrontery, and a mendacity which is sometimes almost incomprehensible. We do not desire to enter into any discussion as to the equality of the white and black races; but in making a comparison of their characters, we conceive that it will be generally acknowledged that the black are superior in the above respects. There are, of course, noble exceptions to the rule; but, in general terms, it is as much the physiological state of a negro to disregard the truth, as it is for an Indian to be uncertain in his ideas in regard to *meum* and *tuum;* the beautiful character of "Uncle Tom" to the contrary notwithstanding. Another element of success would be a profound ignorance which it would be impossible for a light complexioned quack to attain. Strange as it may seem to the uninitiated, you know, kind reader, that this is a most important element of success. It is necessary for

---

cians sued the Union Médicale for having asserted that "homœopathy was neither a doctrine, nor a science, but a trade," and that "if an epoch had ever presented itself at which the method of Hahemann could be employed by any one who was not abjectly ignorant—a crack-brained visionary, or a wretched charlatan—it was certainly not the present one." The editors and proprietors of the Union Médicale pleaded, by way of defence and justification, that what they had stated was only the truth. The tribunal before which the suit was brought, without passing any judgment on the rival claims of allopathy, yet held that the plaintiffs had no ground of action, and dismissed the case, with costs.

a person to be profoundly ignorant of the simplest principles of medicine, in order to take any stand in the ranks of the fraternity of quacks. Without that, how could the practitioner state that a certain disease in a child was due to "an absorbtion of calomel by the system from the flesh of an ox which had had a disease of the skin for which, *perhaps*, mercurial ointment had been applied and had *struck in;*" or that an induration on the finger produced by sewing, was "dependent upon a disease of the medium nerve?" This last *bon mot* was related to us by a patient only a few days since.

In both these qualifications, however, a white man might possibly equal the negro, though such a case would be only the rare exception; but there is a quality in which he would always be his inferior, namely, in external decorations. The natural admirable taste in dress of the black race, with which we are all so familiar, must have a tremendous effect upon the Parissian; and if the reader will return to the paragraph which we have quoted, he will see that this point is particularly mentioned. With all these qualifications, we will have to look no farther than the Parisian negro for our *beau-ideal* of a quack.

This we imagine is nearly as hard a case as we ever have in this country, and we cannot but suppose that it is but one of numerous instances; but look for a moment how the profession of France rid themselves of such an evil, while we submit to it from a necessity which arises from a person's right, in this country, to do what he pleases with his life and health. We copy the following from the Nashville Journal, with the editorial comment:

*Quackery in France.—The Physicians Demand Protection of the Government.—Their Petition to the Emperor.*

*Sir:*—At an epoch in which the history of your benevolence is written in ineffaceable characters on the face of France; at the moment in which society, imitating your example, is striving to ameliorate the condition of the poor classes, the medical corps deem it proper to call the attention of your Majesty to one of the social evils which paralyze your generous intentions.

Sir, alongside of that medicine which consoles and cures, there boldly marches an ignorant, sordid, and illegal medicine.

If, in the cities, the instruction of the masses can sometimes counteract its grievous effects, it is not so in the country, where unfortunately ignorance still predominates, and where it lavishes its scandalous and deceitful promises with unrestrained publicity.

Sir, there is in this a serious danger, which barrier constantly impedes that impulsion which you so freely give to morality and humanity.

Permit us to hope that your Majesty, so careful of the children of the Poor, and so provident for the aged poor, will deign to pursue his work by protecting the whole community by an efficacious *repression* against the illegal practice of medicine.

Such, sir, are the wishes of the medical corps, so often called to the honor of propagating the benefits of your touching solicitude for the suffering, but too often fettered by prejudice in the accomplishment of its mission of humanity.

We have the honor to be, with the most profound respect, the very humble and the very devoted servants of your Majesty. [SIGNATURES.]

The above is a copy of the petition of the deputies of the Medical Societies of the Seine to the Emperor, who received it with great complaisance, and even with gratification. The Emperor expressed surprise that the practice of medicine was not more effectually protected by existing laws, and enquired if it were possible that remedies were advertised in the public journals, and sold to the public, without having received the sanction of the Academy of Medicine; and he promised that the petition should be sent to the proper Minister, with special instructions to examine it and give it the attention that its importance demanded.

Such a measure is impossible in this country; we might petition the legislature year after year without any result; and should we succeed in having any remedy attempted, we would, perhaps, be in as bad a situation as the profession of Massachusetts, who succeeded in procuring the passage of a law, by which irregular practitioners could not collect their fees in the courts. Quacks, however, still found their dupes, whom they compelled to pay in advance, saying with a great deal of force, that it was absolutely necessary, as they could no longer collect by law. This gave this class of practitioners such an advantage, that the profession actually petitioned for the repeal of the law which they had formerly desired, and were content to let the matter rest as before.

We fear that it is impossible for us to put a legal check upon quackery in this country; even the abortionists are permitted to advertise their nefarious compounds, and we fear will continue to do so in spite of us. The articles n our September and October numbers, by which we, in our enthusiastic nexperience, expected to do some good, met with so faint a response from journals and societies, that we reluctantly have given up the battle. We cannot but hope, however, that though our feeble efforts did so little, the powerful influence of the American Medical Association will do much in behalf of the cause. The mind of the profession will be admirably prepared for the subject, by a series of articles which have been commenced by Dr. Storer, of Boston, in the "*North American Medico-Chirurgical Review*." Dr. Storer was directed to prepare a report on this subject, at the meeting of the Association at Nashville, 1857, but has not been able till now to fulfill the task. We wish him all success, and are still convinced that a united action will do some good.

We have expended so much on the Parisian negro, that we have little space left for quacks in general. Where, by the way, do we draw the line? Are we not to regard the homœopathist in that light? We certainly are sufficiently abused, not only by homœopathic practitioners, but by those who employ them, to justify us, when speaking of them at all, to give the only rational view of their pretended system.

Yes, they are a variety of the genus quack, and should be regarded as such, and invariably treated as such by us! We are members of a noble and a dignified profession; one which has grown, by the accumulated labors of great men, to a science, our reverence of which must increase with every advance we make in its mysteries. As members of such a profession, we must countenance in no way the insolent upstarts who seek to rob it of its glory, who traffic in death, and who grow fat upon the spoils wrung from the sufferer deluded by their false promises of health and strength. It is undoubtedly the fact, that irregular practitioners derive most of their gains from persons whose minds, worn down by an incurable malady, and dissatisfied that they can extract no promise of cure from the conscientious physician, morbidly craving encouragement, will go to the quack who promises a perfect cure in a month, for $100, for example, *in advance.* This is the way in which money is extracted by our itinerant doctors, who find it necessary to migrate as soon as there is any danger of a falsification of their promises.

It seems to us that we have but one duty to perform in regard to irregular practitioners, and that that duty is due, not to ourselves, but to the profession which we have chosen. That is, never to countenance them in any way; never to consult with them, to satisfy a patient, for example; never to let the world say that there exists between us and any irregular, merely a friendly difference of opinion in certain points. We may differ in opinion with men with whom we are working for the advancement of a common cause, and acknowledge that we may be wrong and they right; but that is not the case with men who wish to throw discredit upon a profession, which, I hope, we all love with all our soul, and to decry the great names of those whom we cannot but revere. Let us guard our science above all things and do nothing which can take from its dignity, whether we be influenced by the head, or by the heart.

